# Protective effects of calorie restriction on insulin resistance and islet function in STZ-induced type 2 diabetes rats

**DOI:** 10.1186/s12986-021-00575-y

**Published:** 2021-05-05

**Authors:** Li Zhang, Ying-juan Huang, Jia-pan Sun, Ting-ying Zhang, Tao-li Liu, Bin Ke, Xian-fang Shi, Hui Li, Geng-peng Zhang, Zhi-yu Ye, Jianguo Hu, Jian Qin

**Affiliations:** 1 Department of Traditional Chinese Medicine, The Seventh Affiliated Hospital, Sun Yat-Sen University, Shenzhen, 518107 China; 2 Department of Traditional Chinese Medicine, The First Affiliated Hospital, Sun Yat-Sen University, Guangzhou, 518100 China; 3 Department of VIP Ward, Sun Yat-Sen University Cancer Center, Guangzhou, 510080 China; 4 Department of Obese and Metabolic Disease, Guangzhou Panyu Hospital of Chinese Medicine, Guangzhou, 511400 China

**Keywords:** Calorie restriction, Diabetes, Insulin resistance, Streptozotocin, AKT /AS160/GLUT4

## Abstract

**Background:**

Caloric restriction (CR) has become increasingly attractive in the treatment of type 2 diabetes mellitus (T2DM) because of the increasingly common high-calorie diet and sedentary lifestyle. This study aimed to evaluate the role of CR in T2DM treatment and further explore its potential molecular mechanisms.

**Methods:**

Sixty male Sprague–Dawley rats were used in this study. The diabetes model was induced by 8 weeks of high-fat diet (HFD) followed by a single dose of streptozotocin injection (30 mg/kg). Subsequently, the diabetic rats were fed HFD at 28 g/day (diabetic control) or 20 g/day (30% CR regimen) for 20 weeks. Meanwhile, normal rats fed a free standard chow diet served as the vehicle control. Body mass, plasma glucose levels, and lipid profiles were monitored. After diabetes-related functional tests were performed, the rats were sacrificed at 10 and 20 weeks, and glucose uptake in fresh muscle was determined. In addition, western blotting and immunofluorescence were used to detect alterations in AKT/AS160/GLUT4 signaling.

**Results:**

We found that 30% CR significantly attenuated hyperglycemia and dyslipidemia, leading to alleviation of glucolipotoxicity and thus protection of islet function. Insulin resistance was also markedly ameliorated, as indicated by notably improved insulin tolerance and homeostatic model assessment for insulin resistance (HOMA-IR). However, the improvement in glucose uptake in skeletal muscle was not significant. The upregulation of AKT/AS160/GLUT4 signaling in muscle induced by 30% CR also attenuated gradually over time. Interestingly, the consecutive decrease in AKT/AS160/GLUT4 signaling in white adipose tissue was significantly reversed by 30% CR.

**Conclusion:**

CR (30%) could protect islet function from hyperglycemia and dyslipidemia, and improve insulin resistance. The mechanism by which these effects occurred is likely related to the upregulation of AKT/AS160/GLUT4 signaling.

**Supplementary Information:**

The online version contains supplementary material available at 10.1186/s12986-021-00575-y.

## Introduction

Type 2 diabetes mellitus (T2DM) is a metabolic disorder, the occurrence and progression of which are closely related to individual lifestyles and other risks, such as genetic factors [1]. Caloric restriction (CR), the only approved scientific method that can slow aging, has been proven to improve insulin resistance (IR) and islet dysfunction. Moreover, a recent clinical study [2] suggested the potential of a very low-calorie diet for the remission of T2DM. However, the molecular mechanisms underlying CR in T2DM treatment remain unclear.

The insulin signaling pathway is crucial for normal insulin action. In skeletal muscle or white adipose tissue, activated AKT phosphorylates the 160-kDa substrate of AKT (AS160) to block the inactivation of small Rab GTPase protein switches that control vesicle trafficking. This process facilitates the translocation of glucose transporter 4 (GLUT4), further mediating glucose uptake in these tissues to maintain glucose homeostasis [3]. Notably, apart from insulin-induced glucose uptake, most of the other effects of insulin are retained or only slightly affected in individuals with IR. IR can be defined as a decrease in insulin-induced glucose uptake [3]. Therefore, normal transduction of AKT/AS160/GLUT4 signaling in skeletal muscle or white adipose tissue is critical to guarantee well-balanced glucose uptake to maintain normal insulin sensitivity. It has been reported that the phosphorylation level of AKT in patients with IR decreased by up to 50% when compared with that in healthy controls [4–6]. Muscle and adipose tissue-specific GLUT4 knockout can induce global IR and hyperinsulinemia [7, 8]. Mutations in AS160 also lead to IR and an increased risk of progression to T2DM in humans [9].

Based on the aforementioned information, AKT/AS160/GLUT4 signaling may play an important role in the onset of IR and T2DM. Fortunately, the positive effects of CR on AKT and its phosphorylation have been observed in many studies [10–12], and several preliminary studies have reported the potential of CR in regulating GLUT4 [13, 14]. Thus, we speculated that the mechanism underlying the metabolic effects of CR in T2DM treatment may be relate to its regulation of AKT/AS160/GLUT4 signaling. T2DM model rats were induced by feeding high-fat diet (HFD) combined with a single low dose of streptozotocin (STZ) injection, and then maintained on a 30% CR regimen to test our hypotheses.

## Methods and materials

### Materials

Gansulin-R was purchased from Tonghua Dongbao Pharmaceutical Co. (Beijing, China). STZ and all other chemicals were purchased from Sigma-Aldrich (St. Louis, MO, USA) unless stated otherwise. Cell Counting Kit-8 (CCK-8) was purchased from Dojindo Laboratories (Kumamoto, Japan). The rat insulin ELISA assay was purchased from Mercodia (Mercodia AB, Uppsala, Sweden). The primary and secondary antibodies used in the experiments are summarized in table (Additional file 1: Table 1).

### Experimental procedures

#### Animals

A total of 60 weaned and specific-pathogen-free SD rats (male, 6–8 weeks, 180–190 g) were obtained from the Experimental Animal Center of Sun Yat-sen University (Guangzhou, China). The animal experiments were performed according to internationally followed ethical standards and approved by the research ethics committee of the School of Public Health, Sun Yat-sen University (no. 2019-001). Rats were kept on a 12 h light/dark cycle in a temperature-controlled room maintained at (24 ± 1) °C. with a relative humidity of (50 ± 5)% and were maintained on a standard chow diet. Rats were habituated to the conditions for 1 week before modeling.

#### Induction of diabetes

With reference to the method widely used in the existing literature [15–17], rats were fed HFD for 8 weeks and then given a single low-dose of injection of STZ (30 mg/kg) to induce the T2DM model. Specifically, rats were randomly allocated to the blank control group (n = 20) and the model group (n = 40) according to their body mass. Rats in the blank control group had a free normal diet (ND, Guangdong Medical Laboratory Animal Center, Foshan, China; containing 58% carbohydrates, 18% protein, 4.5% fat, and 4% essential vitamins and trace elements, with a total of 3.45 kcal/g). Rats in the model group were fed HFD (Guangdong Medical Laboratory Animal Center, Foshan, China; containing 53.6% normal chow, 15% sucrose, 10% lard, 20% protein, 1.2% cholesterol, and 0.2% sodium cholate, with a total of 4 kcal/g). After 8 weeks of feeding, rats in the model group were intraperitoneally injected with 1% STZ dissolved in 0.1 M citric acid buffer (pH = 4.2–4.5) at a single dose of 30 mg/kg, while the blank control rats received the same amount of citric acid buffer (0.1 M). It is important to note that light had to be avoided throughout the entire procedure of preparation and injection of STZ solution, and the dissolved STZ solution was used as quickly as possible (within 15 min). After STZ administration, model rats were provided with HFD ad libitum in order to attain a relatively stable blood glucose level. Fourteen days after STZ injection, random blood glucose (RBG) was measured with a portable glucometer (Beijing Yicheng biotechnology co. LTD, China) through the tail vein. Rats with RBG levels higher than or equal to 16.7 mmol/L were considered as diabetic [18] and were further used in the following experiment.

#### CR procedure

**Fig. 1:**
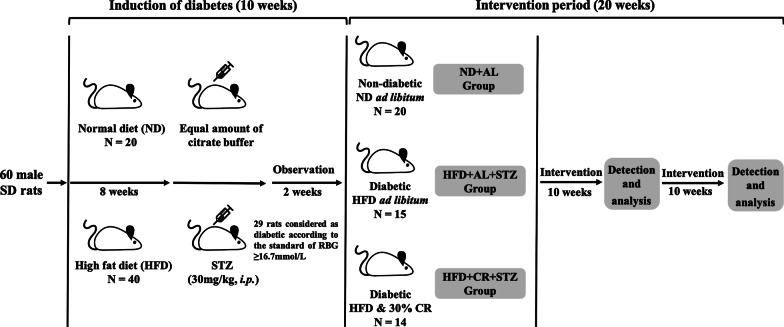
Flow chart of animal study design

During HFD feeding, the total food intake of rats in each cage was recorded to calculate the average daily food intake per rat, which was 28.54 ± 4.78 g/day. Thus, 30% CR (20 g/day) was used in the following experiments. The diabetes model was successfully induced in a total of 29 rats after STZ administration. Thereafter, according to blood glucose level, diabetic rats were randomly assigned to the model control group provided HFD ad libitum with high-fat diet (HFD + AL + STZ, MCT group, n = 15) or the CR intervention group fed a HFD with a 30% CR regimen, as stated above (HFD + CR + STZ, CR group, n = 14). Meanwhile, rats in the blank control group continued to be provided free access to normal diet (ND + AL, BCT group, n = 20). The intervention period lasted for 20 weeks. Water was provided to all rats ad libitum throughout the experiment. The entire process of this experiment is detailed in Fig. 1.

Body mass and water intake were recorded every week, while RBG and fasting blood glucose (FBG) levels were measured every 2 or 4 weeks respectively. To assess insulin resistance, and insulin tolerance test (ITT) was conducted at baseline before modeling, after modeling, and at 10 and 20 weeks post-treatment. In addition, FBG and fasting insulin (FINS) were also detected at the indicated time points through the tail vein after a 12-h overnight fasting period, and insulin levels were measured by ELISA according to the manufacturer’s instructions. An intraperitoneal glucose tolerance test (IPGTT) and glucose-stimulated insulin secretion (GSIS) were performed after modeling (before intervention), and at 10 and 20 weeks after intervention to evaluate glucose tolerance and islet secretion.

At 10 weeks and 20 weeks after intervention, 6–10 rats in each group were anesthetized with 2% pentobarbital sodium (60 mg/kg) after overnight fasting (12 h), and then blood, liver, pancreas, skeletal muscle, and white adipose tissue (WAT, specifically epididymal fat pad) were rapidly collected. Blood samples were centrifuged at 4 °C and 3500 rpm for 15 min to collect the serum and stored at − 80 °C until use. The lipid profile was measured in the central laboratory of the First Affiliated Hospital of Sun Yat-sen University.

#### IPGTT

Rats were intraperitoneally injected with 50% glucose solution (2 g/kg) after overnight fasting for 12 h. Blood glucose was measured before injection (0 min) and 15, 30, 60, 90, and 120 min after injection through the tail vein with a portable glucometer. Six rats from each group were randomly selected for the study.

#### ITT

Rats were intraperitoneally injected with Gansulin-R (0.5 IU/kg) after fasting for 6 h. Blood glucose was measured before injection (0 min) and 15, 30, 45, 60 and 90 min after injection through the tail vein with a portable glucometer. Six rats from each group were randomly selected for the study.

#### GSIS

Rats were intraperitoneally injected with 50% glucose solution (2 g/kg) after 12 h of fasting at night. Blood samples were collected from the tail vein before injection (0 min) and 15, 30 and 60 min after injection. The blood samples were centrifuged at 3500 rpm, 4 °C for 15 min. The separated serum was collected and the insulin level was detected by ELISA. Six rats from each group were randomly selected for the study.

#### Glucose uptake in isolated soleus

At the end of the experiment, glucose uptake in the isolated soleus was measured in four rats from each group. The effect of CR on glucose uptake was detected according to the previous studies, with slight modifications [19–21]. Generally, the isolated fresh soleus was immediately rinsed with Krebs–Ringer buffer, and the muscle fascia, tendons and attached connective tissue were removed as much as possible. Subsequently, the muscle tissue was placed into the brain slice mold (Reward, Shenzhen, China) to cut it into thin slices of similar size with a blade, then divided into three parts (roughly the same weight) and placed into separate 2 mL Eppendorf tubes. The muscle tissue was then incubated with 1.5 mL oxygenated glucose-free Krebs–Henseleit buffer (KRH) containing 0.1% bovine serum albumin (BSA) and 10 mU/mL insulin (Gansulin-R) at 37 °C and 300 rpm (shaking) for 60 min to deplete intercellular glucose. After that, the liquid was removed, oxygenated with KRH (1.5 mL, containing 11.1 mM glucose, 0.1% BSA, and 40 mM mannitol) with 0, 10, or 20 mU/mL insulin. Another tube contained an equal volume of oxygenated KRH, but no muscle tissue served as a blank control. All the four tubes were incubated at 37 °C and 300 rpm (shaking) for 30 min. A 1 mL aliquot was collected from each incubation tube before and after incubation, and the glucose concentration was determined. Glucose uptake of muscle was calculated as the amount of glucose (mg) taken up per gram of muscle tissue using the following formula: muscle glucose uptake = (GC_**b**_ − GC_**a**_)/ muscle tissue weight, where GC_**b**_ and GC_**a**_ are the glucose concentrations before and after incubation, respectively.

#### Immunofluorescence

Skeletal muscle from three rats in each group was randomly selected for GLUT4 immunofluorescence experiments. Briefly, after fixation in 4% paraformaldehyde overnight at 4 °C, the muscle tissues were equilibrated in 20% sucrose overnight at 4 °C until the tissues settled to the bottom. The tissues were then transferred into 30% sucrose overnight at 4 °C until they settled to the bottom, after which they were embedded in OCT for frozen sectioning. Sections were sliced at a thickness of 10 μm. Subsequently, frozen sections were rinsed gently with 4 °C phosphate-buffered saline (PBS) three times, fixed with 4 °C 4% PFA for 30 min at room temperature, and then washed three times with PBS again. After incubation with 0.2% Trition-X100 in PBS for 20 min, sections were blocked with 3% BSA for 30 min at room temperature and then incubated overnight with rabbit anti-GLUT4 antibody (1:250, Abcam, ab654, Cambridge, MA, USA) at 4 °C in a wet box. After washing with Tris-buffered saline containing 0.1% Tween 20, the sections were incubated with donkey secondary antibody conjugated with Alexa Fluor® 568 (1:400, Invitrogen, A-11011, Carlsbad, CA, USA) for 3 h at room temperature. Nuclei were stained with 4′,6-diamidino-2-phenylindole (DAPI, 1 μg/mL, Sigma-Aldrich, St. Louis, MO, USA). All photomicrographs were taken using an inverted fluorescence microscope (Nikon Eclipse Ti-E, Nikon, Japan).

#### Western blotting

Total protein extracts of skeletal muscle and epididymal fat pad tissues were obtained by homogenizing and lysing with RIPA buffer (Beyotime, Shanghai, China), followed by centrifugation at 14,000×*g* for 5 min at 4 °C. Protein concentration was measured using a BCA assay kit (Beyotime, Shanghai, China). Equal amount of protein (20 μg) were loaded into each lane. Proteins were separated by sodium dodecyl sulfate–polyacrylamide gel electrophoresis and electrically transferred to a polyvinylidene difluoride membrane (Millipore, Boston, MA, USA). After blocking the membrane with 5% skim milk, the target proteins were immunodetected using specific antibodies. After incubation with the secondary antibody, bands were visualized using an ECL plus kit (ThermoFisher, Waltham, MA, USA) and exposed to autoradiographic films according to the manufacturer’s instructions. The intensities of bands were determined using ImageJ 1.52q software.

### Statistical analysis

Statistical analysis was performed using SPSS 23.0 software. Data are presented as the mean ± standard deviation (SD), and statistical significance was determined using one-way analysis of variance (ANOVA) followed by multiple comparisons with Tukey's test. Statistical significance was set at *p* < 0.05.

## Results

A total of 40 rats received STZ injection after 8 weeks of HFD feeding, among which 29 attained targeted RBG levels (≥ 16.7 mmol/L) and were used in the following experiment. Five rats died within 3 days of STZ administration because of intolerance to STZ toxicity. Furthermore, RBG concentrations of another 6 rats fluctuated in the range of 5.5–9.8 mmol/L, and were therefore excluded from the following experiment. During the entire intervention period, there were no death in the blank control group (ND + AL). However, two rats in the MCT group (HFD + AL + STZ) died within 6 weeks, probably due to higher blood glucose levels (fluctuated in 22–27 mmol/L) and lower body weight, which influenced their acquisition of food and water. One rat in the CR group (HFD + CR + STZ) died accidentally from excessive anesthesia with isopentane inhalation when drawing blood from the tail vein in the GSIS experiment.

### Effects of CR on body mass and glucolipotoxicity

**Fig. 2:**
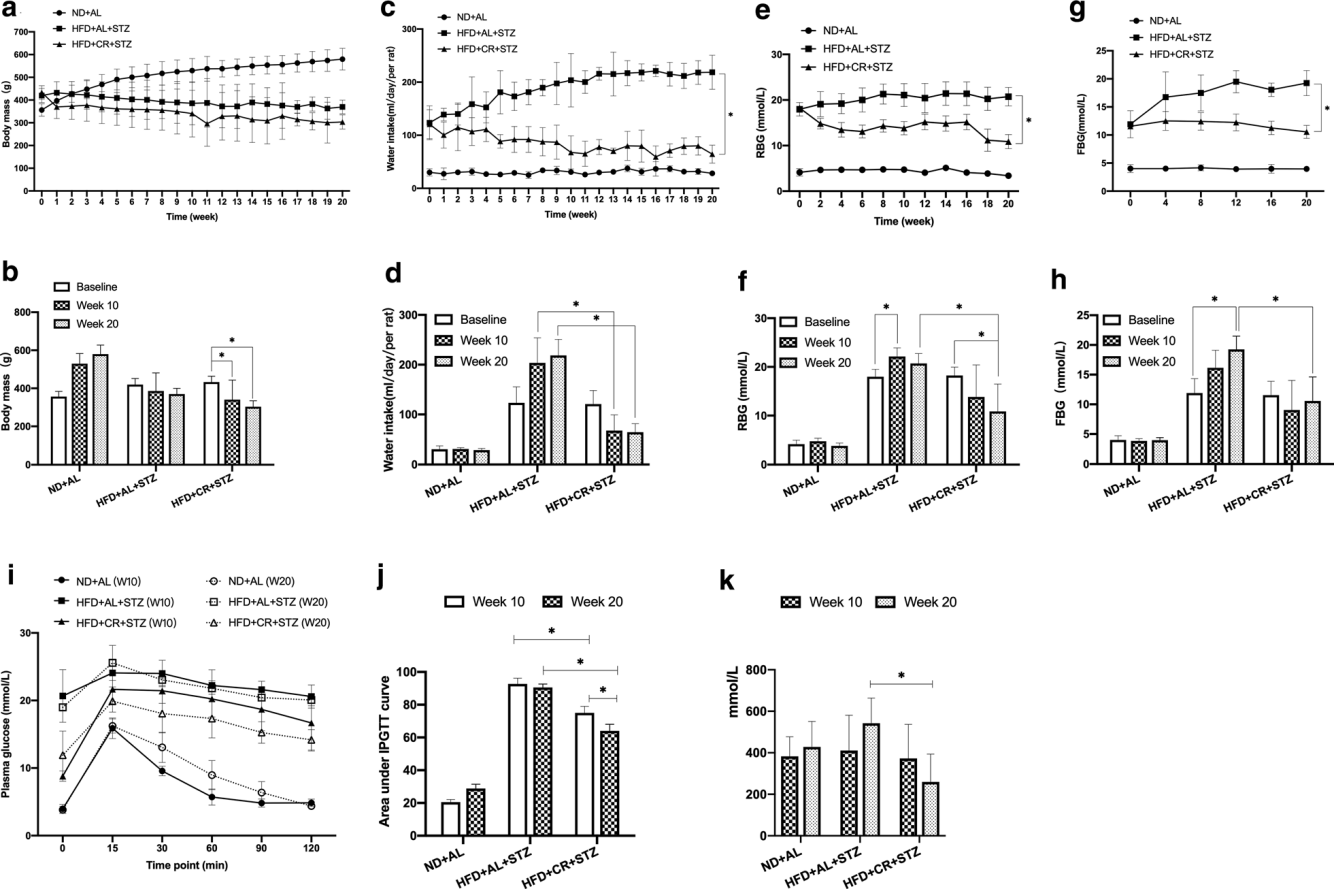
Effects of CR on metabolic indices. The diabetic Sprague–Dawley rats model was induced by feeding HFD for 8 weeks, followed by a single STZ injection (30 mg/kg). The rats were either provided HFD *ab libitum* (HFD + AL + STZ group) or fed HFD with a 30% restriction regimen (HFD + CR + STZ group). Rats given free access to normal chow diet served as a blank control (ND + AL group). All interventions lasted for 20 weeks. **a**, **b** body mass; **c**, **d** water intake; **e**, **f** RBG; **g**, **h** FBG; **i** IPGTT; **j** AUC of IPGTT; **k** FFAs. Data were presented as the mean ± standard deviation (SD). For **a–h**, n = 10–20 for the ND + AL group and n = 6–14 for the other two groups. Six rats from each group were randomly chosen for the IPGTT experiment and lipid profile detection. *p < 0.05 between the two groups; CR, calorie restriction; HFD, high-fat diet; STZ, streptozotocin; RBG, random blood glucose; FBG, fasting blood glucose; IPGTT, intraperitoneal glucose tolerance test; AUC, area under curve; FFAs, free fat acids; ND + AL, normal diet provided ad libitum (BCT group); HFD + AL + STZ, HFD provided ad libitum and STZ injection (MCT group); HFD + CR + STZ, HFD with 30% CR and STZ injection (CR group)

Body weight at each time point in the CR group was lower than that in the MCT group, but the difference was not statistically significant (Fig. 2a, b, *p* > 0.05). However, when compared with the baseline, a significant decrease was observed in the CR group (Fig. 2a, b, *p* < 0.05) but not in the MCT group (Fig. 2a, b, *p* > 0.05). Meanwhile, water intake in the MCT group increased gradually, while it declined to the blank control level after 20 weeks of CR intervention (Fig. 2c, d, *p* > 0.05).

During the intervention period, RBG and FBG levels increased significantly in the MCT group and decreased gradually in the CR group. Ultimately, hyperglycemia was ameliorated by 20 weeks of CR intervention. (Fig. 2e, f, *p* < 0.05). Meanwhile, improvement in lipid profile including free fatty acids (FFAs, Fig. 2k, *p* < 0.05) was also detected after CR intervention (Additional file 1: Fig. 1). These results demonstrated that CR intervention provide relief from glucolipotoxicity. Accordingly, we found that glucose tolerance in the CR group at 20 weeks was significantly improved when compared to that at baseline or in the MCT group. (Fig. 2i, j, *p* < 0.05). The area under the curve (AUC) of the IPGTT also showed a similar variation tendency (Fig. 2j, *p* < 0.05).

### Effects of CR on insulin resistance and islets function

**Fig. 3:**
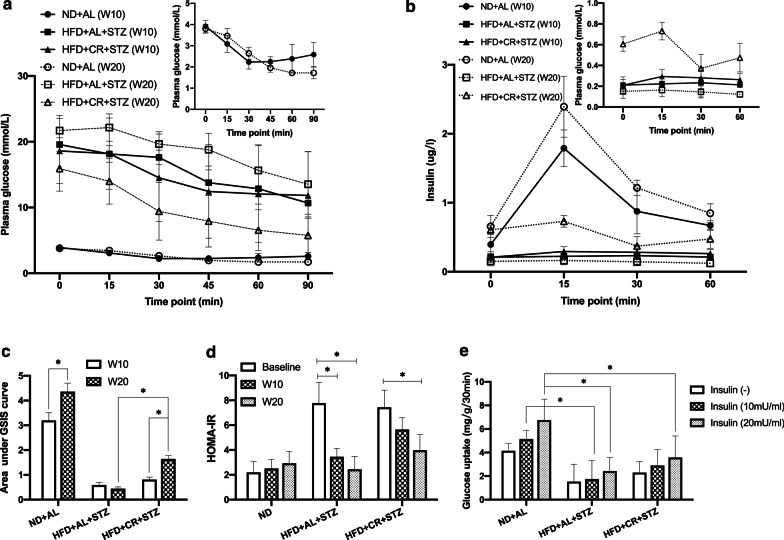
Effects of CR on insulin resistance and islets function. The diabetic Sprague–Dawley rats model was induced by feeding HFD for 8 weeks, followed by a single STZ injection (30 mg/kg). The rats were either provided HFD *ab libitum* (HFD + AL + STZ group) or fed HFD with a 30% restriction regimen (HFD + CR + STZ group). Rats given free access to normal chow diet served as a blank control (ND + AL group). All interventions lasted for 20 weeks. **a** ITT (n = 6); **b** GSIS (n = 6); **c** AUC of GSIS; **d** HOMA-IR (n = 6–10); **e** glucose uptake in muscle (n = 4); data were presented as the mean ± standard deviation (SD). *p < 0.05 between the two groups; CR, calorie restriction; HFD, high-fat diet; STZ, streptozotocin; ITT, insulin tolerance test; GSIS, glucose simulate insulin secretion; AUC, area under curve; HOMA-IR, homeostasis model assessment of insulin resistance; IOD, integrated optical density; ND + AL, normal diet provided ad libitum (BCT group); HFD + AL + STZ, HFD provided ad libitum and STZ injection (MCT group); HFD + CR + STZ, HFD with 30% CR and STZ injection (CR group)

After 20 weeks of intervention, insulin sensitivity was notably improved in the CR group compared with that in the MCT group, as indicated by the ITT results. (Fig. 3a, *p* < 0.05). Interestingly, homeostatic model assessment for insulin resistance (HOMA-IR) values in the CR and MCT groups both exhibited a significant decreasing tendency, and were significantly lower than their baseline values (Fig. 3d, *p* < 0.05). Glucose- stimulated insulin secretion levels in the CR and MCT groups were both much lower than those in the BCT group (Fig. 3b, c, *p* < 0.05). Nevertheless, GSIS gradually improved in the CR group and progressively deteriorated in the MCT group over time (Fig. 3b, c). Insulin levels and AUC of GSIS in the CR group at 20 weeks were significantly higher than those in the baseline and the MCT group, although they were still markedly lower than those in the BCT group (Fig. 3b, c, *p* < 0.05).

### Effects of CR on glucose uptake in skeletal muscle

The glucose uptake rate of muscle in the BCT group increased proportionally with the elevation of insulin concentration applied for stimulation, while this effect was distinctly impaired in both the CR and MCT groups (Fig. 3e). Although the glucose uptake rate in the CR group at each insulin concentration was slightly higher than that in the MCT group, the difference was not statistically significant (Fig. 3e, *p* > 0.05).

### Effects of CR on AKT/AS160/GLUT4 signaling

**Fig. 4:**
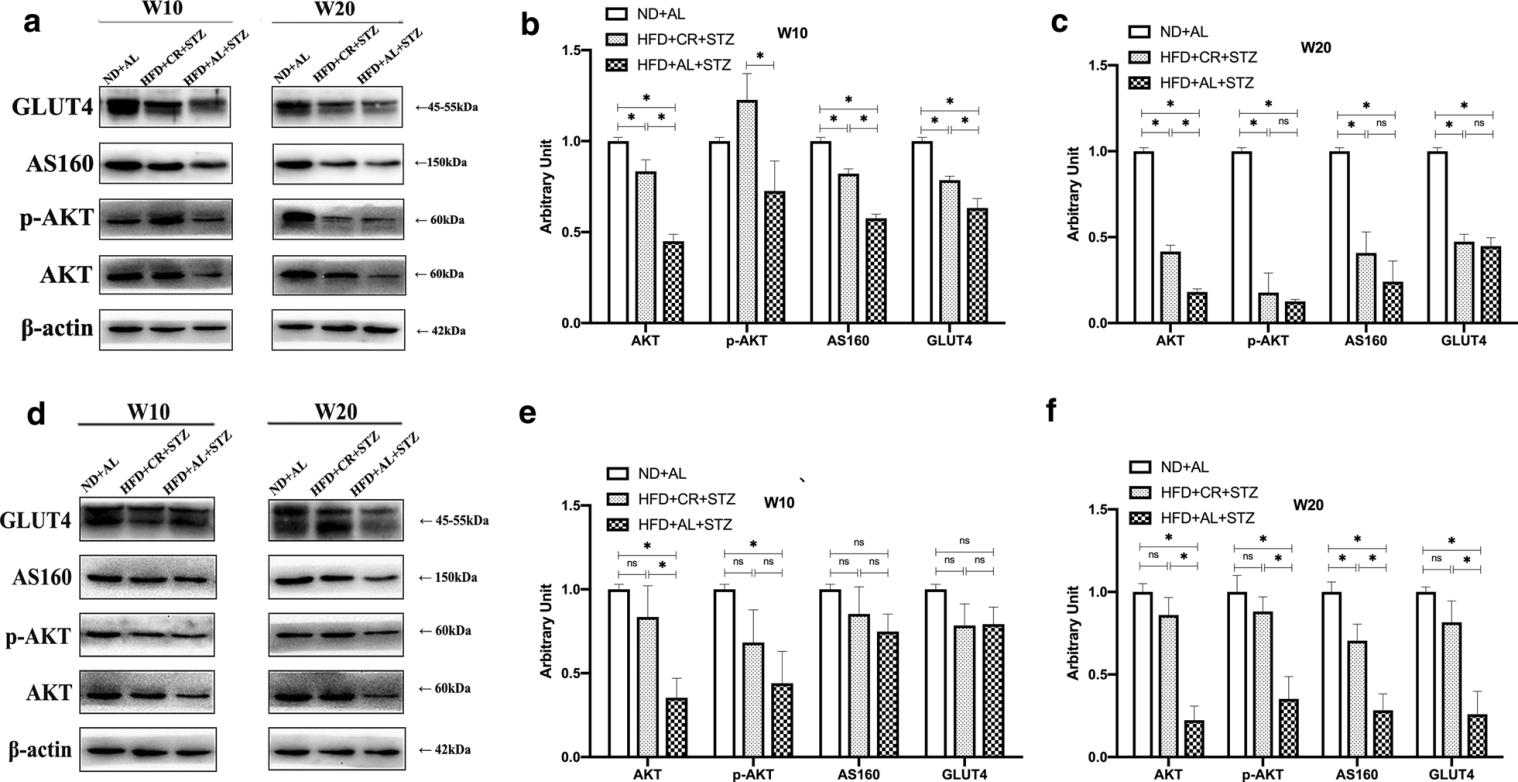
Effects of CR on AKT/AS160/GLUT4 signaling. The diabetic Sprague–Dawley rats model was induced by feeding HFD for 8 weeks, followed by a single STZ injection (30 mg/kg). The rats were either provided HFD *ab libitum* (HFD + AL + STZ group) or fed HFD with a 30% restriction regimen (HFD + CR + STZ group). Rats given free access to normal chow diet served as a blank control (ND + AL group). All interventions lasted for 20 weeks. Effects of CR on the expressions of AKT, p-AKT, AS160, and GLUT4 were determined by western blotting. **a** Expressions of AKT, p-AKT, AS160 and GLUT4 in skeletal muscle tissue after 10 and 20 weeks of intervention. **b**, **c** Densitometric measurements of band intensity were performed using ImageJ 1.52q. **d** Expressions of AKT, p-AKT, AS160 and GLUT4 in white adipose tissue after 10 and 20 weeks of intervention. **e**, **f** Densitometric measurements of band intensity were performed using ImageJ 1.52q. Data were from at least three independent experiments and presented as the mean ± standard deviation (SD). *p < 0.05 between the two groups; CR, calorie restriction; HFD, high-fat diet; STZ, streptozotocin; ND + AL, normal diet provided ad libitum (BCT group); HFD + AL + STZ, HFD provided ad libitum and STZ injection (MCT group); HFD + CR + STZ, HFD with 30% CR and STZ injection (CR group)

**Fig. 5:**
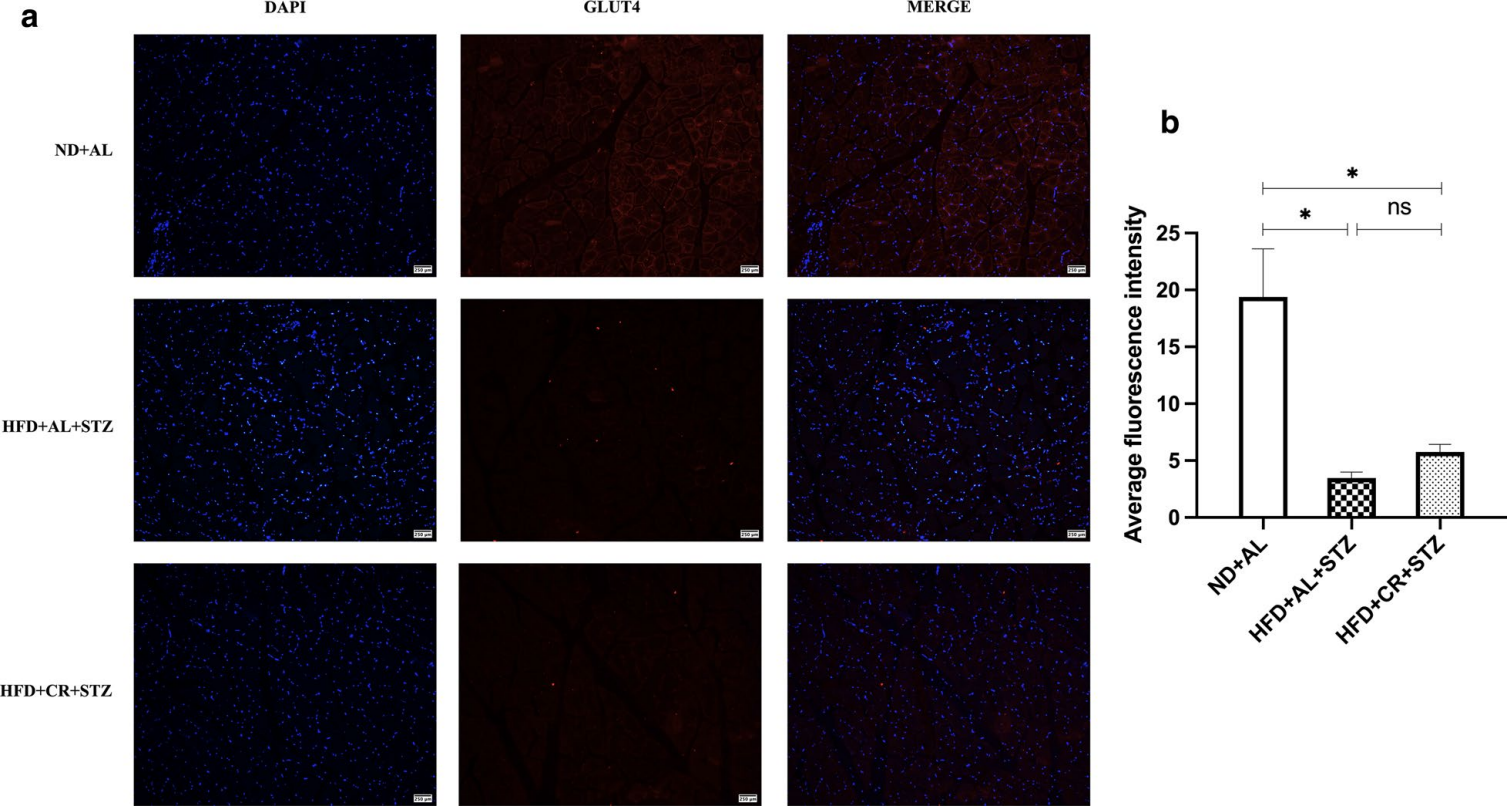
GLUT4 immunofluorescence in skeletal muscle. The diabetic Sprague–Dawley rats model was induced by feeding HFD for 8 weeks, followed by a single STZ injection (30 mg/kg). The rats were either provided HFD *ab libitum* (HFD + AL + STZ group) or fed HFD with a 30% restriction regimen (HFD + CR + STZ group). Rats given free access to normal chow diet served as a blank control (ND + AL group). All interventions lasted for 20 weeks. **a** Representative images of GLUT4 immunofluorescence in skeletal muscle (× 100). **b** Densitometric measurements of the average fluorescence were performed by ImageJ 1.52q. Data were from at least three independent experiments and presented as the mean ± standard deviation (SD). *p < 0.05 between the two groups; CR, calorie restriction; HFD, high-fat diet; STZ, streptozotocin; ND + AL, normal diet provided ad libitum (BCT group); HFD + AL + STZ, HFD provided ad libitum and STZ injection (MCT group); HFD + CR + STZ, HFD with 30% CR and STZ injection (CR group)

In skeletal muscle, CR intervention could exert protective effects on the greatly reduced expression of AKT, p-AKT, AS160, and GLUT4 proteins induced by T2DM pathology and HFD feeding. However, the protective effects of CR wore off over time. The expression of the aforementioned proteins and the average fluorescence intensity of GLUT4 decreased to the same levels as the MCT group at 20 weeks (Fig. 4a–c, Fig. 5, *p* > 0.05). In contrast, in white adipose tissue, the protein expression of AKT, p-AKT, AS160, and GLUT4 in the MCT group decreased sharply over time, and were markedly lower than those in the CR groups at 20 weeks (Fig. 4d–f, *p* < 0.05).

## Discussion

In a diabetic rat model induced by HFD feeding combined with a single low-dose injection of STZ, we demonstrated that 30% CR could protect islet function against glucolipotoxicity during the progression of T2DM by markedly relieving hyperglycemia and dyslipidemia. CR also improved insulin resistance significantly, with probable underlying molecular mechanisms related to the upregulation of AKT/AS160/GLUT4 signaling.

CR has attracted increasing attention in the treatment of metabolic diseases, as overeating and obesity are increasingly common in modern society. Therefore, weight loss has always been considered as the main effect of CR and the source of its other accompanying metabolic benefits. However, some studies [22, 23] have indicated that weight loss may not be the only key function of CR in improving glucose metabolism. In our study, hyperglycemia in MCT rats deteriorated over time, resulting in aggravated osmotic diuresis, massive loss of glucose, and subsequent continuous weight loss. In contrast, diabetic rats receiving CR intervention experienced great relief from hyperglycemia, protecting them from continuous weight loss. However, insufficient calorie intake might offset the effect of weight gain resulting from hyperglycemia relief, and may eventually lead to a slight (but not significant) decline in body mass when compared with MCT rats.

Glucotoxicity refers to long-term chronic hyperglycemia leading to downregulated insulin gene expression and chronic irreversibly decreased insulin synthesis. Meanwhile, lipotoxicity refers to the toxic effect of high concentrations of fatty acids in circulation on β cells [24]. In our study, hyperglycemia was constantly aggravated in the MCT group over time, indicating more and more severe glucotoxicity. Accordingly, the results from GSIS also revealed almost exhausted insulin secretion. Fortunately, 30% CR markedly reversed the deterioration of hyperglycemia, leading to relief from glucotoxicity and thus protection of islet function. The role of CR in improving the lipid profile has been widely demonstrated in previous studies [25, 26], as well as in this study. High FFAs level is an independent risk factor for T2DM [27, 28]. In vitro studies have shown that FFAs can damage glucose-stimulated insulin secretion in β cells and primary islets, and increase β-cell apoptosis and necrosis [29, 30]. Our data demonstrated that sharply increased FFAs level induced by HFD feeding and T2DM pathology could be reversed by 30% CR. The alleviation of lipotoxicity seemed to exert positive effects on β cells as GSIS was eventually improved after CR intervention in our study.

The insulin signaling pathway is crucial for normal insulin action. Defects in the transduction or phosphorylation of the amongst signaling molecules such as PI3K, AKT, and GLUT4 may be relate to the onset of IR. Our data indicated that AKT/AS160/GLUT4 signaling in skeletal muscle was consecutively downregulated with the progression of diabetes and HFD feeding, while CR intervention seemed to exert limited protective effects. Although the expression of AKT, p-AKT, AS160, and GLUT4 proteins was restored to some extent by 30% CR at 10 weeks, their expression declined to the levels observed in MCT rats at 20 weeks. In accordance with this, the improvement in glucose uptake induced by 30% CR was not significant. These results probably suggest that the overall IR improvement induced by 30% CR may not be due to the alleviation of IR in muscle. This needs to be further confirmed in the future studies. An in vitro study [31] found no significant change in basal glucose uptake rate of skeletal muscle tissue treated with medium containing glucose and insulin equivalent to the CR level in vivo when compared with the control group. Mechanism analysis detected no notable changes in p-AKT, AS160, GLUT1, and GLUT4, which was similar to our results. In visceral white adipose tissue, the protein expression levels of AKT, p-AKT, AS160 and GLUT4 in the MCT group decreased progressively over time, which was consistent with the downregulation of insulin signaling in IR individuals reported by many studies. However, these effects could be significantly reversed by 30% CR intervention, suggesting the potential of 30% CR in delaying impairment of insulin signaling in white adipose tissue during the process of T2DM.

In addition, it was suggested [32] that the remaining β cells may proliferate after the destruction of STZ, resulting in spontaneous recovery from hyperglycemia. Hence, diabetic rats were fed HFD to maintain model stability in our experiment. However, overt glucolipotoxicity induced by consecutive HFD feeding accelerated islet failure, especially in the MCT group. Then, the decrease in FINS was much greater than the increase in FBG; therefore, the HOMA-IR value of the MCT group calculated with the formula also exhibited a decreasing tendency, contrary to the aggravated IR reflected by the ITT curve.

Admittedly, the implementation of CR with HFD due to the instability of the STZ-induced diabetic model may have greatly weakened the protective effects of CR. This is one of the limitations of our research. In addition, we did not include included more rats or other diabetic animal models, such as transgenic models (*db*/*db*, *ob*/*ob),* to verify the results repeatedly. In the future, transgenic animal models and larger sample sizes should be employed to better interpret the benefits of CR. The protective effects of CR under the condition of blocked AKT/AS160/GLUT4 signaling have not been explored yet, and further studies are warranted to consolidate our findings.

## Conclusions

In conclusion, our study demonstrated that during the progression of T2DM, 30% CR could exert protective effects on islet function through significant alleviation of glucolipotoxicity, reflected by markedly improved hyperglycemia and dyslipidemia, and ameliorate insulin resistance. The underlying molecular mechanism are likely related to the upregulation of AKT/AS160/GLUT4 signaling in white adipose tissue. Further studies associated with of AKT/AS160/GLUT4 signaling need to be conducted to enhance the credibility of our results.

## Supplementary Information

**Additional file 1:** Antibodies used in the experiments and changes in lipid profile post-treatment.

## Data Availability

The datasets used and/or analyzed during the current study are available from the corresponding author upon reasonable request.

## Abbreviations

T2DM: Type 2 diabetes mellitus
CR: Calorie restriction
HFD: High-fat diet
IR: Insulin resistance
INSR: Insulin receptor
IRS: Insulin receptor substrate
PI3K: Phosphoinositide-3-kinase
AKT: Protein kinase B
AS160: AKT substrate of 160 kDa
GLUT4: Glucose transporter 4
STZ: Streptozotocin
RBG: Random blood glucose
FBG: Fasting blood glucose
ITT: Insulin tolerance test
FINS: Fasting insulin
HOMA-IR: Homeostasis model assessment of insulin resistance
IPGTT: Intraperitoneal glucose tolerance test
GSIS: Glucose-stimulated insulin secretion
WAT: White adipose tissue
KRH: Krebs–Henseleit buffer
BSA: Bovine serum albumin
PBS: Phosphate-buffered saline
FFAs: Free fatty acids
TG: Triglyceride
TC: Total cholesterol
HDL-c: High-density lipoprotein cholesterol
LDL-c: Low-density lipoprotein cholesterol
