# Supplementary material for: Protective effects of calorie restriction on insulin resistance and islet function in STZ-induced type 2 diabetes rats
Source: Nutr Metab (Lond). 2021 May 5;18:48. doi: 10.1186/s12986-021-00575-y (PMC8097947; doi:10.1186/s12986-021-00575-y)
Supplement: Supplementary file 1 — Additional file 1: Antibodies used in the experiments and changes in lipid profile post-treatment. [file 12986_2021_575_MOESM1_ESM.docx]

**Supplementary material**

**Table 1**

**Summary of antibodies and working conditions used in western blotting**

| **Antibodies** | **Species** | **Source** | **Dilution** |
| --- | --- | --- | --- |
| **Primary antibodies** |  |  |  |
| Anti-β-actin | Rabbit, Polyclonal | Invitrogen, Carlsbad, CA, USA | 1:1000 |
| Anti-AKT | Rabbit, Monoclonal | Cell Signaling Technology, Beverly, MA, USA | 1:1000 |
| Anti-p-Akt (Ser473) | Rabbit, Monoclonal | Cell Signaling Technology, Beverly, MA, USA | 1:2000 |
| Anti-AS160 | Rabbit, Polyclonal | Affinity Bioscience, Cincinnati, OH, USA | 1:1000 |
| Anti-GLUT4 | Rabbit, Polyclonal | Abcam, Cambridge, MA, USA | 1:2000 |
|  |  |  |  |
| **Secondary antibody** |  |  |  |
| Anti-Rabbit IgG(HP) | Rabbit, Polyclonal | GE Healthcare, Chicago, IL, USA | 1:30000 |


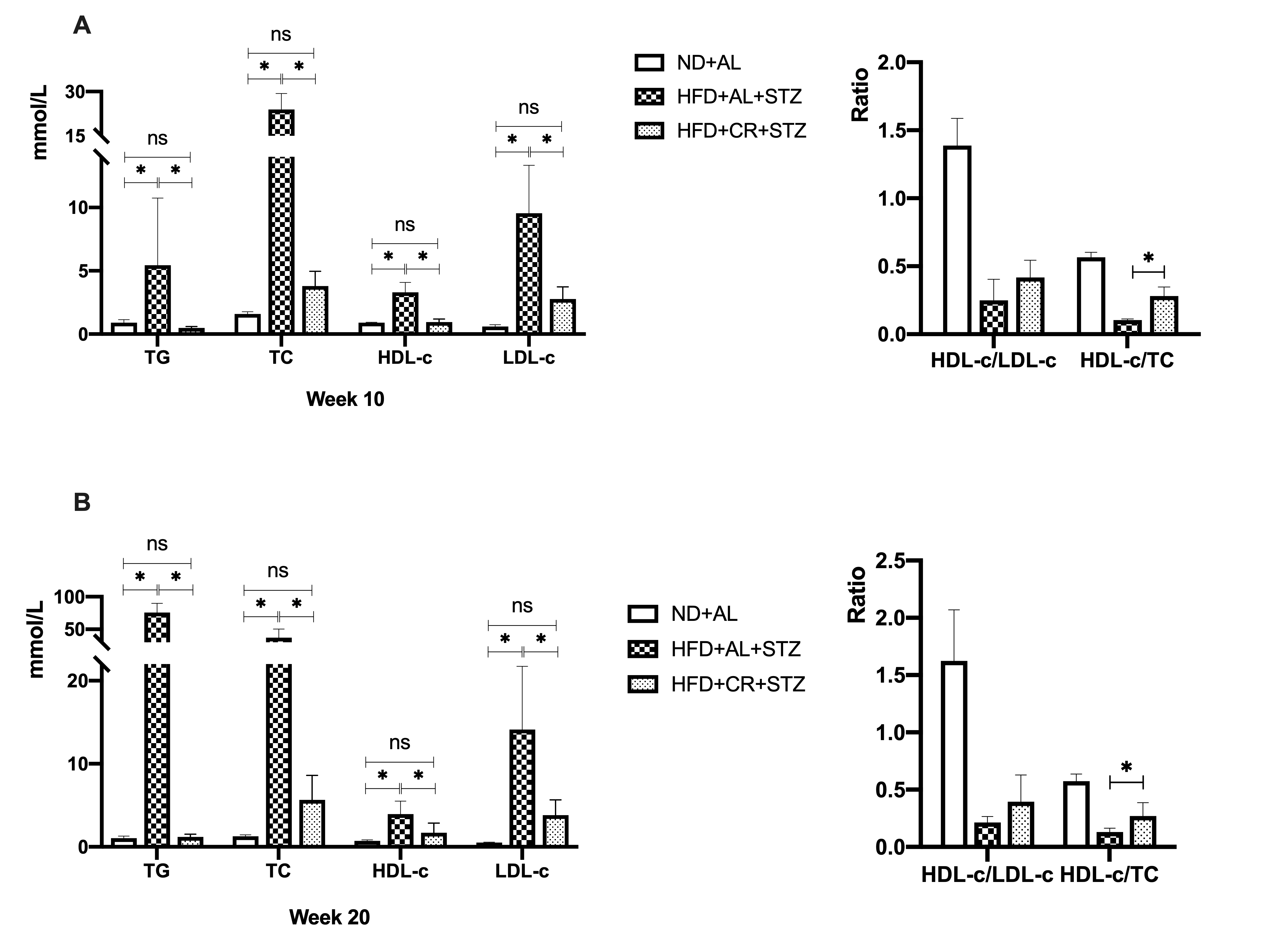


**Fig. 1 Effects of CR on lipid profile**

The diabetic Sprague-Dawley rats model was induced by feeding HFD for 8 weeks, followed by a single STZ injection (30 mg/kg). The rats were either provided HFD *ab libitum* (HFD + AL + STZ group) or fed HFD with a 30% restriction regimen (HFD + CR + STZ group). Rats given free access to normal chow diet served as a blank control (ND + AL group). All interventions lasted for 20 weeks. **(A)** and **(B)** Lipid profiles after 10 and 20 weeks of treatment. Data were presented as mean ± S.D. n=6 for each group. * *p* < 0.05 between the two groups; **CR**, calorie restriction**; TG**, triglyceride; **TC**, total cholesterol; **HDL-c**, high-density lipoprotein cholesterol; **LDL-c**, low-density lipoprotein cholesterol. **CR**, calorie restriction; **HFD**, high-fat diet; **STZ**, streptozotocin; **ND + AL**, normal diet provided ad libitum (BCT group); **HFD + AL + STZ**, HFD provided ad libitum and STZ injection (MCT group); **HFD + CR + STZ**, HFD with 30% CR and STZ injection (CR group).
